# Seroprevalence of viral and bacterial pathogens among malaria patients in an endemic area of southern Venezuela

**DOI:** 10.1186/s40249-023-01089-w

**Published:** 2023-04-10

**Authors:** David A. Forero-Peña, Fhabián S. Carrión-Nessi, Mary Lopez-Perez, Marisol Sandoval-de Mora, Iván D. Amaya, Ángel F. Gamardo, Melynar Chavero, Luisamy Figuera, María V. Marcano, Natasha A. Camejo-Ávila, Mariana Hidalgo, Cariagne J. Arenas, Myriam Arévalo-Herrera, Sócrates Herrera

**Affiliations:** 1 Biomedical Research and Therapeutic Vaccines Institute, Ciudad Bolivar, Venezuela; 2 Internal Medicine Department, “Ruiz y Páez” University Hospital Complex, Ciudad Bolivar, Venezuela; 3 “Dr. Francisco Battistini Casalta” Health Sciences School, University of Oriente – Bolivar Nucleus, Ciudad Bolivar, Venezuela; 4 Department of Immunology and Microbiology, Faculty of Health and Medical Sciences, Centre for Medical Parasitology, University of Copenhagen, Copenhagen, Denmark; 5 Immunoparasitology Laboratory, Microbiology and Cell Biology Centre, Venezuelan Institute for Scientific Research, Miranda, Venezuela; 6 Caucaseco Scientific Research Centre, Cali, Colombia

**Keywords:** Malaria, Seroprevalence, Co-infection, Chikungunya, Dengue, Hepatitis, Venezuela

## Abstract

**Background:**

Malaria remains a leading public health problem worldwide. Co-infections with other pathogens complicate its diagnosis and may modify the disease’s clinical course and management. Similarities in malaria clinical presentation with other infections and overlapping endemicity result in underdiagnosis of co-infections and increased mortality. Thus, the aim of this study was to determine the seroprevalence of viral and bacterial pathogens among diagnosed malaria patients in malaria-endemic areas in Venezuela.

**Methods:**

A cross-sectional study was conducted on malaria patients attending three reference medical centres in Ciudad Bolivar, Venezuela. Clinical evaluation and laboratory tests for dengue virus (DENV), chikungunya virus (CHIKV), viral hepatitis [hepatitis A virus (HAV), hepatitis B virus (HBV), and hepatitis C virus (HCV)], and leptospirosis (LEP) were performed by enzyme-linked immunosorbent assays. Previous exposure to these pathogens was defined by the presence of specific immunoglobulin (Ig) G, and co-infection or recent exposure (CoRE) was determined by the presence of specific IgM alone or IgM + IgG. Data analysis considered descriptive statistics. Parameter distribution was statistically evaluated using Kolmogorov–Smirnov test and the necessary comparison tests. Odds ratio (*OR*) for complications was determined according to CoRE presence with a 95% confidence interval (*CI*).

**Results:**

A total of 161 malaria patients were studied, 66% infected with *Plasmodium vivax*, 27% with *P. falciparum*, and 7.5% harboured *P. vivax*/*P. falciparum* mixed infection. Previous exposure to DENV (60%) and CHIKV (25%) was frequent. CoRE was confirmed in 55 of the 161 malaria patients (34%) and were more frequent in *P. falciparum* (49%) than in *P. vivax* (29%) and mixed malaria patients (25%) (*OR* = 2.43, 95% *CI*: 1.39–4.25, *P* = 0.018). The most frequent CoRE was DENV (15%), followed by HAV (12%), HBV (6.2%), CHIKV (5.5%), and LEP (3.7%); HCV CoRE was absent. Complicated malaria was significantly more frequent in patients with CoRE (56%) than those without CoRE (36%; *OR* = 2.31, 95% *CI*: 1.18–4.92, *P* = 0.013).

**Conclusions:**

We found high CoRE prevalence in malaria patients as determined by serology in the study region; cases were associated with a worse clinical outcome. Further prospective studies with samples from different infection sites and the use of molecular tools are needed to determine the clinical significance of these findings.

**Graphical abstract:**

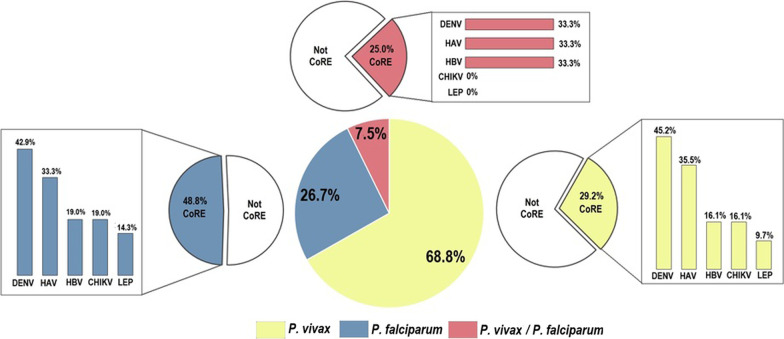

**Supplementary Information:**

The online version contains supplementary material available at 10.1186/s40249-023-01089-w.

## Background

Malaria remains a leading public health problem worldwide, with an estimated 247 million cases and 619,000 deaths in 84 malaria-endemic countries in 2021 [1]. In America, malaria cases have been reduced by 60% between 2000 and 2021, but several countries in the region have shown extraordinary progress. Argentina, El Salvador, and Paraguay have been declared malaria-free by the World Health Organisation (WHO), and countries in Central America such as Haiti have experienced a reduction of more than 40% since 2015. In contrast, Venezuela experienced a dramatic increase from 35,500 cases in 2000 to over 467,000 cases and 403 deaths reported in 2019 [2]; these figures represent more than half of the malaria clinical cases and over 70% of the deaths reported in the region [3]. In 2021, *Plasmodium vivax* accounted for 82% of cases in Venezuela, followed by *P. falciparum* (13%) and *P. vivax*/*P. falciparum* mixed infection (4%) [1]; historically, 70–80% of malaria cases in Venezuela have been reported in Bolivar state [4].

Malaria is often associated with outdoor occupations, including mining and farming, exposing people to other vector-borne diseases. Although it is often assumed that fever is due only to malaria in malaria-endemic countries, there is evidence of widespread over-diagnosis in people presenting with severe febrile illness [5]. However, in recent years an unexpected increase and spread of dengue virus (DENV), chikungunya virus (CHIKV) [6, 7], leptospirosis (LEP) [8], and viral hepatitis [hepatitis A virus (HAV), hepatitis B virus (HBV), and hepatitis C virus (HCV)] [9, 10], among others have been reported in malaria endemic regions [11]. This suggests the need to emphasise differential diagnoses of febrile cases in tropical countries with malaria-endemic areas, mainly in unexpected clinical findings or apparent inadequate responses to antimalarial drugs.

Infections with more than one pathogen complicate the diagnosis and modify the clinical course of the diseases and their management. In addition, similarities in the clinical presentation of malaria and these arboviruses and icteric febrile diseases, combined with overlapping endemicity, may result in the underdiagnosis of co-infections. Despite limited information on the clinical outcome and the precise interactions of these pathogens in co-infections, it is known that multiple infections confound the malaria course. Diagnosis of non-malaria febrile illness in neglected regions remains a challenge, and delay in diagnosis or treatment initiation for any of these infections could have fatal outcomes [12]. In rural malaria-endemic areas, diagnostic tests for confirmation of other diseases are scarce and demand more infrastructure. Even when available, such tests have a short window to detect the pathogen. In both cases, detecting pathogens other than malaria is difficult to confirm, leaving the serological confirmation of recent or past patients’ contact with such pathogens as an alternative.

The recent decline of the Venezuelan health system capacity has led to the deterioration of the epidemiological surveillance and malaria control program [2]. Additionally, Venezuela’s tropical location favours the co-circulation of different zoonoses, including arboviruses, and, consequently, the presence of multiple co-infections in malaria patients. We describe here a cross-sectional study conducted to determine the seroprevalence of viral and bacterial pathogens among patients diagnosed with malaria at the three main diagnostic centres of Ciudad Bolivar, capital of Bolivar state, Venezuela.

## Methods

### Study sites

Venezuela, located on the northern coast of South America, has a surface area of ~ 916,445 km^2^. Bolivar state is its largest state, covering an extension of ~ 240,500 km^2^ and located in the south of the country, bordering Brazil and Guyana, with an approximate population of 1,837,485 inhabitants distributed among 11 municipalities and 46 parishes [13]. Most of the population resides in the northernmost and easternmost parts of the state, where economic activities are related to mining (iron, gold, bauxite, and diamonds), hydroelectric industries, businesses and services, forestry, cattle raising, and agricultural development.

The total number of accumulated malaria cases in Venezuela between 2007 and 2017 was 1,207,348 (range: 32,037–411,586), with overall malaria incidence rates (cases/1000 inhabitants per year) increasing from 5.2 (2007) to 28 (2017). Bolívar contributed ~ 47% of the total cases in Venezuela during 2017, but during the previous years this region accounted for 60–80% of malaria reported for the whole country. *P. vivax* is the most prevalent parasite accounting for 70–80% of malaria cases, whereas *P. falciparum* causes the remaining cases, with a mean ratio of *P. vivax/P. falciparum* of 3.04 [4].

This study was conducted in Ciudad Bolivar, the Bolivar state capital, with approximately 427,399 inhabitants [13]. Three reference centres for the care, diagnosis, and treatment of malaria patients were selected: “El Perú” and “La Sabanita” health centres, and “Ruiz y Páez” University Hospital Complex. “El Perú” and “La Sabanita” are type-II urban outpatient health centres which attend most (~ 90%) of the cases over 12 years of age in the city, whereas the “Ruiz y Páez” University Hospital Complex, a type-IV hospital, is the main hospitalisation centre of Ciudad Bolivar.

Regarding the epidemiological behaviour of other viral and bacterial pathogens in Venezuela, mainly in Bolivar state, the information is limited and outdated. The average incidence of dengue between 2010 and 2016 was 211 cases per 100,000 inhabitants. Within the country, the temporal increase in dengue cases reflects the national dengue incidence, with the highest incidence in the most densely populated regions (central regions) and those bordering Colombia and Brazil (border regions) [2]. The total number of chikungunya cases in Venezuela reported by the Pan American Health Organisation in 2014 was 34,945, with an incidence of 121.5 per 100,000 inhabitants [14]. The actual prevalence in Venezuela of leptospirosis in humans is unknown; however, the Regional Epidemiology Directorate of the Bolivar State Public Health Institute reported morbidity rates of 1.02 and 1.0 per 100,000 inhabitants for 1998 and 1999, respectively [15]. Finally, a time series analysed the incidence rate of viral hepatitis during the period from 1990 to 2016 documenting an average rate of 20.9, 4.4, and 0.9 per 100,000 inhabitants for HAV, HBV, and HCV, respectively; unfortunately, rates by state are not available [16].

### Study design and patients

A cross-sectional study was conducted from June to November 2018. Individuals over 12 years of age and of both sexes with malaria-compatible symptoms and confirmed diagnosis by microscopic examination of thin and thick blood smears, regardless of parasite species and origin, were invited to participate in the study. Trained physicians at each study site performed a detailed physical examination of all study participants according to the local standard of care. Clinical manifestations, socio-demographic information, diagnosis, and treatment of patients were recorded on a standard evaluation form. Blood samples were collected by venepuncture from each patient once diagnosis was confirmed, and patients provided informed consent upon a detailed explanation of the study objectives and methods, followed by antimalarial treatment by the local healthcare provider using the national antimalarial protocol (approved in 2017) [17]. Patients were classified as having complicated or non-complicated malaria according to the WHO [18] and *Ministerio del Poder Popular para la Salud* of Venezuela criteria [17], regardless of the malaria parasite species.

### Blood samples and laboratory tests

Blood samples (8 ml) drawn from each patient by arm venepuncture were used for haematological analysis, blood chemistry tests (urea, creatinine, glycemia, electrolytes, transaminases, and lactate dehydrogenase), and serological tests against DENV, CHIKV, HAV, HBV, HCV, and LEP. All the laboratory tests were performed once at the local private laboratory “Centro Especializado de Investigación Clínica 42” (Ciudad Bolivar). Enzyme-linked immunosorbent assays (ELISA) were used to determine specific immunoglobulin (Ig) M (IgM) and IgG antibodies to DENV (BQ Kits, Inc., San Diego, CA, USA), CHIKV (Abcam, Waltham, MA, USA), HAV (Abcam, Waltham, MA, USA), and LEP (SERION, Würzburg, Germany), as well as IgM to HCV (Abcam, Waltham, MA, USA), HBV surface antigen, and HBV core antibody (Abcam, Waltham, MA, USA), according to the manufacturer’s instructions.

These serological tests were selected based on sensitivity and specificity, all higher than 92%. Since plasma antibodies against these diseases persist from a few months for IgM to several years for IgG, we defined co-infection or recent exposure (CoRE) as detection of anti-DENV, anti-CHIKV, anti-HAV, anti-HCV, and anti-LEP IgM alone or IgM plus IgG. HBV CoRE was defined as detection of HBV surface antigen. Previous exposure to any of the pathogens was defined as the presence of IgG and absence of IgM. We were not able to confirm co-infection by other techniques.

### Data analysis

Statistical analysis was performed using Statistical Package for the Social Sciences version 25 (International Business Machines Corporation, Armonk, NY, USA), and figures were generated with Microsoft Power BI version 2.78 (Microsoft, Redmond, WA, USA) and the statistical software R 4.0.3 (Lucent Technologies, Jasmine Mountain, USA). Data analysis considered descriptive statistics. The distribution of the parameters was statistically evaluated using Kolmogorov–Smirnov test and the necessary comparison tests were applied. The odds ratio (*OR*) for complications was determined according to the presence of CoRE with a 95% confidence interval (*CI*). A *P*-value < 0.05 was considered statistically significant.

## Results

### Demographic and epidemiological characteristics

A total of 161 patients diagnosed with malaria were included, of whom 106 (65.8%) had *P. vivax*, 43 (26.7%) *P. falciparum*, and 12 (7.5%) mixed malaria (*P. vivax*/*P. falciparum*) infection. Most of the enrolled individuals were male (64%) and younger than 40 years (77%), with no statistically significant differences between the three parasite species groups (*P* = 0.19). Mineworker (37.2%) and housewife (18.6%) were the most frequent occupations. Self-reported previous malaria episodes were common among the study population, ranging from one to eight episodes in lifetime. Previous exposure to DENV (59.6%) and CHIKV (24.8%), as determined by the presence of specific IgG, was frequent in the study samples, with no differences between malaria parasite species. Previous exposure to HBV (6.8%) and LEP (3.7%) was less frequent, and no samples were positive for HAV or HCV-specific IgG. Fifty-five (34.2%) of the patients had CoRE with at least one pathogen. Whereas a single pathogen was identified in most patients (44/55, 80%), more than one pathogen was identified in the remaining 11 patients. The prevalence of CoRE was 14.9%, 11.8%, 6.2%, 5.5%, and 3.7% for DENV, HAV, HBV, CHIKV, and LEP, respectively; no patients were found with HCV. In patients with CoRE with two or more pathogens, simultaneous DENV/HAV was found in 4/11 (36.4%), while other combinations like DENV/CHIKV, HAV/HBV, HAV/LEP, CHIKV/LEP, and DENV/LEP were present in one patient each. We found two patients with CoRE by three pathogens, one with DENV/CHIKV/HAV (male, 34 years old, infected by *P. vivax*, from the Sifontes municipality, miner) and the other with DENV/CHIKV/LEP (male, 30 years old, infected by *P. vivax*, from the Heres municipality, miner).

**Fig. 1:**
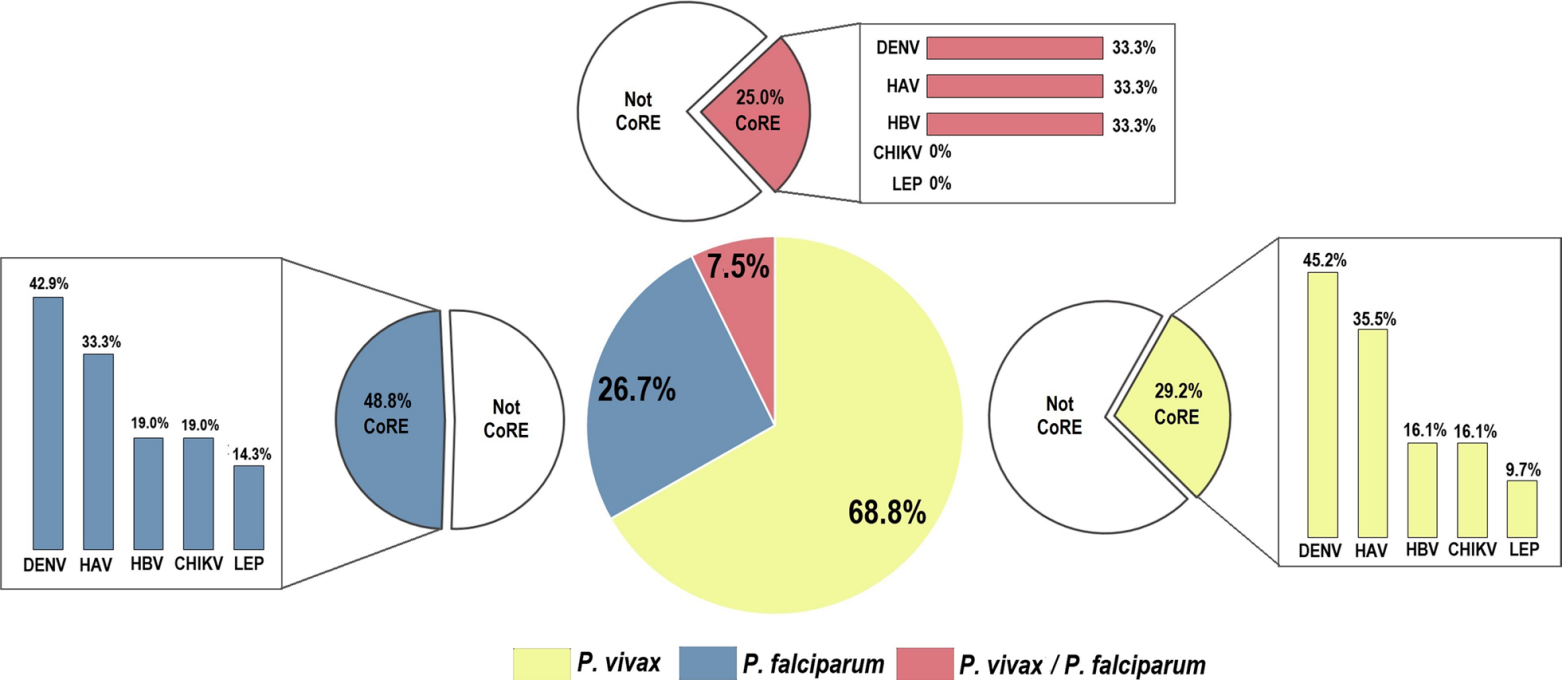
Frequency of CoRE with other pathogens among malaria patients according to parasite species. Central pie chart shows proportions of patients according to *Plasmodium* species. Small pie charts show proportions of patients with CoRE (IgM alone or IgM + IgG). Bars show proportions of patients according to CoRE pathogen. Patients with more than one CoRE were added individually to each group. *CoRE* Co-infected or recently exposed, *DENV* Dengue virus, *HAV* Hepatitis A virus, *HBV* Hepatitis B virus, *CHIKV* Chikungunya virus, *LEP* Leptospirosis

CoRE with at least one pathogen were significantly more frequent in patients with *P. falciparum* (21/43, 48.8%) than with *P. vivax* (31/106, 29.2%) or mixed malaria infection (3/12, 25%; *OR* = 2.43, 95% *CI*: 1.39–4.25, *P* = 0.018; Fig. 1).

As shown in Table 1, the characteristics of the groups were homogeneous, except for a higher frequency of CoRE cases with HAV found in Heres municipality than in other municipalities (*P* = 0.048). Out of the 156 patients from Bolivar state, most (60/156, 38.5%) had been in Sifontes municipality during the last month, mainly in “Kilómetro 88” and “El Dorado” towns. The second most frequent municipality of origin was Heres (52/156, 33.3%), where mainly came from Ciudad Bolivar (Additional file 1). Regarding origin by species, 21/43 (48.8%) of patients with *P. falciparum*, 6/12 (50%) of those with mixed infection, but only 33/106 (31.1%) of patients with *P. vivax* came from the Sifontes municipality. In contrast, 41/106 (38.6%), 9/43 (20.9%), and 2/12 (16.6%) of patients with *P. vivax*, *P. falciparum*, and mixed infection, respectively, came from the Heres municipality. Most of the mineworkers (36.7%) were from the Sifontes municipality.

**Table 1:** Demographic and epidemiological characteristics of malaria-infected patients according to CoRE by other viral or bacterial pathogens

Characteristics	CoRE		DENV (*n* = 24)	HAV (*n* = 19)	HBV (*n* = 10)	CHIKV (*n* = 9)	LEP (*n* = 6)
	No (*n* = 106)	Yes (*n* = 55)					
Age, mean (*SD*), years	34 (13.5)	34 (14.5)	32 (14.9)	35 (14.1)	38 (10.3)	33 (14.1)	38 (17.5)
Sex, *n* (%)							
Female	36 (33.9)	22 (40.0)	11 (45.8)	6 (31.6)	4 (40.0)	4 (44.4)	2 (33.3)
Male	70 (66.1)	33 (60.0)	13 (54.2)	13 (68.4)	6 (60.0)	5 (55.6)	4 (66.7)
Municipality*, *n* (%)							
Heres	33 (31.1)	19 (34.5)	4 (16.7)	10 (52.6)	4 (40.0)	4 (44.4)	2 (33.3)
Sifontes	41 (38.7)	19 (34.5)	11 (45.8)	3 (15.8)	3 (30.0)	4 (44.4)	3 (50.0)
Angostura	13 (12.3)	5 (9.1)	3 (12.5)	1 (5.3)	1 (10.0)	–	–
Caroni	1 (0.9)	2 (3.6)	2 (8.3)	2 (10.5)	–	–	–
Cedeño	2 (1.9)	2 (3.6)	1 (4.2)	1 (5.3)	–	1 (11.1)	–
El Callao	7 (6.6)	2 (3.6)	1 (4.2)	1 (5.3)	–	–	–
Piar	–	2 (3.6)	1 (4.2)	–	1 (10.0)	–	–
Sucre	4 (3.8)	4 (7.3)	1 (4.2)	1 (5.3)	1 (10.0)	–	1 (16.7)
No Bolivar state	5 (4.7)	–	–	–	–	–	–
Education, *n* (%)							
High school	43 (40.6)	32 (58.2)	16 (66.7)	10 (52.6)	4 (40.0)	4 (44.4)	1 (16.7)
Primary	42 (39.6)	16 (29.1)	7 (29.2)	6 (31.6)	3 (30.0)	4 (44.4)	4 (66.7)
Professional	8 (7.5)	2 (3.6)	1 (4.2)	–	1 (10.0)	–	–
Technical	5 (4.7)	2 (3.6)	–	2 (10.5)	1 (10.0)	–	–
Neither	8 (7.5)	3 (5.5)	–	1 (5.3)	1 (10.0)	1 (11.1)	1 (16.7)
Occupation^†^, *n* (%)							
Miner	43 (40.6)	17 (30.9)	9 (37.5)	2 (10.5)	6 (60.0)	3 (33.3)	1 (16.7)
Housewife	19 (17.9)	11 (20.0)	5 (20.8)	6 (31.6)	1 (10.0)	3 (33.3)	1 (16.7)
Merchant	10 (9.4)	2 (3.6)	2 (8.3)	1 (5.3)	–	–	–
Student	4 (3.8)	2 (3.6)	1 (4.2)	1 (5.3)	1 (10.0)	–	–
Farmer	5 (4.7)	5 (9.1)	1 (4.2)	3 (15.8)	1 (10.0)	–	1 (16.7)
Other	22 (20.8)	17 (30.9)	6 (25.0)	5 (26.3)	1 (10.0)	3 (33.3)	3 (50.0)
Unemployed	3 (2.8)	1 (1.8)	–	1 (5.3)	–	–	–

Patients with CoRE with more than one pathogen were added independently to each group. Statistically significant differences in ^*^HAV (*P* = 0.048; Fisher’s exact test), ^†^HAV (*P* = 0.038; Fisher’s exact test) and in patients with CoRE with ^†^HBV (*P* = 0.434; Fisher’s exact test). *CoRE* Co-infection or recent exposure, *DENV* Dengue virus, *HAV* Hepatitis A virus, *HBV* Hepatitis B virus, *CHIKV* Chikungunya virus, *LEP* Leptospirosis. – means there was no patient counting

### Clinical manifestations

The most frequent symptoms in malaria patients were fever (100%), chills (100%), and headache (98.1%), without significant differences between the different groups. CoRE with DENV was associated with asthenia (*P* = 0.025), cough (*P* = 0.033), splenomegaly (*P* = 0.011), and somnolence (*P* = 0.003). CoRE with HBV was associated with lower pallor (*P* = 0.023), while CoRE with HAV was associated with stupor (*P* = 0.038) and seizures (*P* = 0.038) (Table 2).

**Table 2:** Symptoms and signs in malaria patients according to CoRE by other viral or bacterial pathogens

	CoRE		DENV (*n* = 24)	HAV (*n* = 19)	HBV (*n* = 10)	CHIKV (*n* = 9)	LEP (*n* = 6)
	No (*n* = 106)	Yes (*n* = 55)					
*Symptoms*							
Fever	106 (100)	55 (100)	24 (100)	19 (100)	10 (100)	9 (100)	6 (100)
Chill	106 (100)	55 (100)	24 (100)	19 (100)	10 (100)	9 (100)	6 (100)
Headache	103 (97.2)	55 (100)	24 (100)	19 (100)	10 (100)	9 (100)	6 (100)
Lumbar pain	50 (47.2)	23 (41.8)	11 (45.8)	9 (47.4)	3 (30.0)	3 (33.3)	2 (33.3)
Asthenia*	47 (44.3)	33 (60.0)	17 (70.8)	9 (47.4)	3 (30.0)	8 (88.9)	4 (66.7)
Diaphoresis	41 (38.7)	24 (43.6)	10 (41.7)	7 (36.8)	4 (40.0)	6 (66.7)	3 (50.0)
Abdominal pain	38 (35.8)	23 (41.8)	11 (45.8)	9 (47.4)	3 (30.0)	3 (33.3)	3 (50.0)
Myalgias	43 (40.6)	23 (41.8)	13 (54.2)	9 (47.4)	2 (20.0)	3 (33.3)	2 (33.3)
Arthralgia	37 (34.9)	23 (41.8)	12 (50.0)	10 (52.6)	2 (20.0)	4 (44.4)	2 (33.3)
Otalgia^†^	2 (1.9)	5 (9.1)	3 (12.5)	1 (5.3)	–	–	1 (16.7)
Dyspnoea	14 (13.2)	6 (10.9)	5 (20.8)	1 (5.3)	–	2 (22.2)	1 (16.7)
Bleeding	–	2 (3.6)	2 (0.3)	–	–	2 (22.2)	–
Cough^‡^	13 (12.3)	10 (18.2)	7 (29.2)	4 (21.1)	–	–	1 (16.7)
Diarrhoea	14 (13.2)	7 (12.7)	4 (16.7)	2 (10.5)	1 (10.0)	–	–
Emesis	20 (18.9)	11 (20.0)	5 (20.8)	5 (26.3)	1 (10.0)	2 (22.2)	1 (16.7)
*Signs*							
Jaundice	34 (32.1)	16 (29.1)	9 (37.5)	6 (31.6)	1 (10.0)	2 (22.2)	2 (33.3)
Pallor^§^	60 (56.6)	29 (52.7)	15 (62.5)	8 (42.1)	2 (20.0)	6 (66.7)	2 (33.3)
Splenomegaly^||^	5 (4.7)	9 (16.4)	4 (16.7)	2 (10.5)	–	2 (22.2)	1 (16.7)
Hepatomegaly	10 (9.4)	6 (10.9)	10 (9.4)	1 (5.3)	1 (10.0)	2 (22.2)	1 (16.7)
Oliguria	6 (5.7)	7 (12.7)	4 (16.7)	1 (5.3)	1 (10.0)	1 (11.2)	1 (16.7)
Somnolence^¶^	9 (8.5)	8 (14.5)	6 (25)	2 (10.5)	–	2 (22.2)	2 (33.3)
Stupor**	1 (0.9)	2 (3.6)	2 (8.3)	2 (10.5)	–	1 (11.1)	–
Seizures^††^	1 (0.9)	2 (3.6)	2 (8.3)	2 (10.5)	–	1 (11.1)	–
Oedema	3 (2.8)	3 (5.5)	2 (6.7)	1 (5.3)	–	–	–
Ascites	–	1 (1.8)	1 (4.8)	–	–	–	–

Data are expressed as frequency and percentages. Patients with CoRE with more than one pathogen were added independently to each CoRE group. Statistically significant association with *DENV (*P* = 0.025; Pearson’s chi-squared test), ^†^no CoRE (*P* = 0.034; Pearson’s chi-squared test), ^‡^DENV (*P* = 0.033; Fisher’s Exact test), ^§^HBV (*P* = 0.023; Fisher’s exact test), ^||^DENV (*P* = 0.011; Yates’s chi-squared test), ^¶^DENV (*P* = 0.003; Yates’s chi-squared test), ^**^HAV (*P* = 0.038; Yates’s chi-squared test), ^††^HAV (*P* = 0.038; Yates’s chi-squared test). *CoRE* Co-infection or recent exposure, *DENV* Dengue virus, *HAV* Hepatitis A virus, *HBV* Hepatitis B virus, *CHIKV* Chikungunya virus, *LEP* Leptospirosis. – means there was no patient counting

### Laboratory findings

Haemoglobin levels were similar between patients with CoRE and not CoRE. Patients with CoRE by CHIKV had lower leucocyte counts (*P* = 0.010). Patients with CoRE generally showed elevated aspartate aminotransferase (AST) levels, but CoRE with HAV was associated with higher AST levels (*P* = 0.007). Interestingly, we found a significant association between CoRE with LEP and low haematocrit (*P* = 0.047) and platelet counts (*P* = 0.019), elevated AST (*P* = 0.006) and alanine aminotransferase (ALT) (*P* = 0.034) levels, and low potassium levels (*P* = 0.043) (Table 3).

**Table 3:** Paraclinical findings on malaria patients according to CoRE by other viral or bacterial pathogens

Laboratory	CoRE		DENV (*n* = 24)	HAV (*n* = 19)	HBV (*n* = 10)	CHIKV (*n* = 9)	LEP (*n* = 6)
	No (*n* = 106)	Yes (*n* = 55)					
Haemoglobin, g/dL	11 (2)	11 (2)	10 (3)	12 (2)	11(2)	11(2)	10 (2)
Haematocrit, %	35 (5)	33 (7)	32 (9)	3 (7)	34 (4)	33 (9)	29 (7)^*^
Leucocytes, 10^9^/L	6 (2)	6 (4)	7 (5)	6 (2)	6 (1)	5 (1)^†^	6 (2)
Platelets, 10^9^/L	90 (44)	87 (55)	100 (76)	76 (29)	95 (24)	80 (25)	57 (24)^‡^
Glycemia, mg/dL	78 (26)	81 (23)	90 (23)^§^	74 (15)	84 (24)	80 (24)	80 (36)
Urea, mg/dL	32 (22)	40 (33)	22 (44)	32 (9)	38 (33)	37 (20)	38 (21)
Creatinine, mg/dL	1 (0.6)	1.2 (1.2)	1.4 (1.7)	1.1 (0.3)	1 (0.2)	1.3(0.4)	1 (0.2)
TB, mg/dL	3 (2.8)	3.7 (3)	3.6 (3.6)	4.1 (3.2)	4 (3.3)	2.3 (0.8)	3.2 (2.2)
AST, U/L	90 (62)	121 (98)^||^	118 (114)	118 (70)^¶^	89 (44)	136 (77)	190 (148)^**^
ALT, U/L	93 (99)	136 (115)	84 (67)	98 (169)	85 (62)	117 (63)	227 (160)^††^
LDH, U/L	541 (190)	530 (156)	457 (85)	546 (153)	583 (202)	530 (143)	530 (56)
Sodium, mmol/L	140.2 (2.3)	140 (3.8)	139.8 (5.1)	140.5 (2.8)	140.1 (1.6)	139.8 (3.2)	139.5 (5.3)
Potassium, mEq/L	4.2 (0.5)	4.2 (0.6)	4.4 (0.5)	4.4 (0.4)	4.4 (0.5)	4.3 (0.8)	3.5 (0.7)^‡‡^
Chlorine, mEq/L	102 (3)	103 (3)	103 (2.96)	103.1 (2.7)	101.7 (1.73)	104.6 (3.4)	105.7 (5.69)

Data are expressed as mean (*SD*). Patients with CoRE with more than one pathogen were added independently to each CoRE group. Statistically significant difference using Student’s* t*-test: *P = 0.047, ^†^*P* = 0.010, ^‡^*P* = 0.019, ^§^*P* = 0.016, ^||^*P* = 0.023, ^¶^*P* = 0.007, ^**^P = 0.006, ^††^*P* = 0.034, ^‡‡^*P* = 0.043*. CoRE* Co-infection or recent exposure, *DENV* Dengue virus, *HAV* Hepatitis A virus, *HBV* Hepatitis B virus, *CHIKV* Chikungunya virus, *LEP* Leptospirosis, *TB* Total bilirubin, *AST* Aspartate aminotransferase, *ALT* Alanine aminotransferase, *LDH* Lactate dehydrogenase, *SD* Standard deviation

### Association between complicated malaria and CoRE

**Fig. 2:**
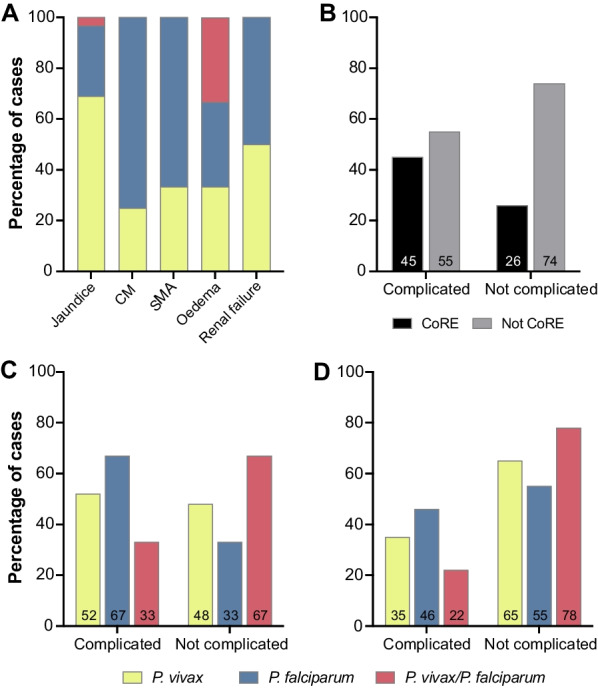
Malaria complications and CoRE. **A** Percentage of cases according to the malaria parasite species. **B** Frequency of CoRE according to the malaria clinical status. Frequency of *Plasmodium* species by malaria clinical status among (**C**) patients with CoRE: *P. vivax* (*P* = 0.42, Pearson’s chi-squared test), *P. falciparum* (*P* = 0.23, Pearson’s chi-squared test), mixed malaria (*P* = 0.82, Pearson’s chi-squared test), and (**D**) patients with not CoRE: *P. vivax* (*P* = 0.69, Pearson’s chi-squared test), *P. falciparum* (*P* = 0.29, Pearson’s chi-squared test), mixed malaria (*P* = 0.6, Yates’s chi-squared test). *CM* Cerebral malaria, *SA* Severe anaemia, *PO* Pulmonary oedema, *CoRE* Co-infected or recently exposed

A total of 69 (42.9%) patients were classified as having complicated malaria. Of those, 42 (60.8%) were caused by *P. vivax*, 24 (34.7%) by *P. falciparum*, and 3 (4.3%) by mixed malaria infection. As expected, the proportion of complicated cases over total cases per parasite species was higher for *P. falciparum* than for *P. vivax* cases and for mixed malaria infection (55.8% vs 39.6% vs 25%, respectively; *P* = 0.045). Eight patients (11.6%) had two or more complications simultaneously. Taken individually, the most frequent complications were jaundice (plasma or serum bilirubin > 3 mg/dL; 84.1%), cerebral malaria (11.6%), severe anaemia (8.7%), pulmonary oedema (4.4%), and renal failure (2.9%) (Fig. 2A). Complicated malaria was significantly more frequent in the CoRE group than in the no CoRE group (56.4% vs 35.8%; *OR* = 2.31, 95% *CI*: 1.18–4.92, *P* = 0.013) (Fig. 2B). No significant differences were found when comparing complications by parasite species within the CoRE group and in the no CoRE group (Fig. 2C, D).

## Discussion

Several studies, mainly from sub-Saharan Africa and Southeast Asia, report *Plasmodium* spp. co-infections or co-circulation with other pathogens, such as DENV [19, 20], CHIKV [21–23], HAV [9], HBV [10, 24], LEP [25, 26], human immunodeficiency virus [27], intestinal helminths [28], and other febrile diseases [29–31]. However, in Latin America, these reports are limited [10, 32–35]. To the best of our knowledge, despite the multiple infections that may complicate malaria and lead to failure in treatment responsiveness, there are no reports on the interactions of these pathogens in co-infections in Venezuela, the most malaria-endemic region in Latin America. This study found a high prevalence of CoRE with other pathogens among malaria patients (34.2%), even higher than that reported in Brazil (20%) [32], but lower than that found in a recent study in India (60%) [36]. Thus, physicians should suspect co-infection in malaria cases with inadequate response to treatment or atypical clinical manifestations.

The prevalence of CoRE with DENV (14.9%) was higher than that found in a large cross-sectional study in hospitalised patients with acute febrile syndrome in the Brazilian Amazon (2.8%) [32], and in a study conducted in Mumbai (India) (10.3%) [19], but lower than in New Delhi (India) (44%) [36] and Pakistan (33.3%) [37]. Thus, the prevalence of co-infection or co-circulation may fluctuate, depending on the local endemicity even within the same country [16, 29], as well as on the sensitivity of the diagnostic methods used. In these studies, prevalence was estimated based on hospitalised and non-hospitalised patients; therefore, it could not be extrapolated to the community level. The high incidence found may also be due to the fact that Venezuela has had the highest dengue incidence rates in the region, along with Brazil and Colombia, as well as a total of six increasingly large nationwide epidemics between 2007 and 2016, compared to four epidemics in the previous 16 years [2].

Malaria CoRE with other pathogens does not induce a predictable clinical pattern. While we found that CoRE with DENV was significantly associated with somnolence and splenomegaly, a study conducted in French Guiana showed a worse clinical outcome, with a higher risk of severe thrombocytopenia and anaemia [35], or induced low platelet counts [37], or elevated transaminases [19]. More surprising is that a study in Peru indicated that *Plasmodium*/DENV co-infection was not associated with disease worsening [33], whereas in another study in India DENV serotype-4 co-infection was associated with even mild malaria [36]. Among the multiple factors that might influence the clinical outcome of the infection, one could hypothesise that differences in DENV serotypes or *Plasmodium* species, previous exposure to either pathogen or to both, age and gender, and other epidemiological conditions may explain the discrepancies among the studies.

The second most frequent CoRE was HAV (11.8%), higher than that found in children from sub-Saharan Africa (1.7%) [9]. This high incidence could be due to a deteriorated water system in Venezuela [38] added to low vaccination rates [39], as confirmed by the very high percentage of individuals (88%) in which HAV-specific IgG was not detected. The age group studied could explain this fact; however, the information on this co-infection is limited. In Venezuela, in the case of hepatitis A vaccination, it is not included in the Venezuelan Expanded Programme on Immunisation, it is only applied in the private sub-sector and in the years of better economic boom, prior to 2013, it reached between 10 and 15% of vaccination coverage, according to personal and press reports [16]. On the other hand, the prevalence of CoRE with HBV (6.2%) in our study was similar to that found in Nigeria (6.6%) [24], but higher than that documented by Braga et al. [34] in western Brazilian Amazon (4.2%). In the same study, patients with co-infection showed no clinical differences from those with only malaria, and similar to our findings also showed no association with classic signs of a liver disorder. In another study, HBV co-infection was more likely to be asymptomatic, even *Plasmodium* parasitaemia correlated inversely with plasma HBV DNA levels [10]. In contrast, other studies revealed that co-infection amongst individuals significantly affected haematological and liver parameters [40, 41]. Our result should be interpreted with caution due to the low sample size. Although co-infection is possible, we found no CoRE with HCV cases [42]. Our finding may explain the low HCV prevalence previously reported in Venezuela [43].

CoRE with CHIKV was found in a lower proportion (5.5%) than that found in Tanzanian (7.1%) [21] and Kenyan children (9.4%) [22]. In contrast, two extensive studies in India [44] and Senegal [45] found a low co-infection prevalence (1.3% and 0.02%, respectively). The observed variations in CHIKV prevalence among different studies may be attributed to epidemiological and geographical factors [46–48]. Our findings on CoRE with LEP (3.7%) contrast with those reported in southern India (22%) [26] and Thailand (7.7%) [49], likely due to the higher incidence of LEP in these regions. A high prevalence (80.6%) of leptospirosis among febrile patients with high suspicion of LEP has been reported in Ciudad Bolivar [15]. Other LEP co-infection cases have been documented [25] and have even been associated with severe sepsis [50]. We found an association between CoRE with LEP with elevated aminotransferases and thrombocytopenia, as previously described [51, 52]. In Latin America, LEP/malaria co-infections are rarely reported, but high clinical suspicion should prevail as late diagnosis could increase morbidity and mortality. Thus, in patients with complicated malaria presenting with fever, thrombocytopenia, and alterations in liver and kidney function, the diagnosis [53, 54] and empirical treatment for LEP co-infection should be considered.

Interestingly, simultaneous CoRE with DENV/HAV was found in four malaria patients. There are few case reports of this concurrent mixed infection [55]. Thus, those are likely to occur more frequently than reported in the available literature, mainly in developing countries. Other CoRE with two or three pathogens could be explained by overlapping breeding sites of mosquito vector species, especially in malaria, DENV, and CHIKV [11, 56]. Additionally, febrile disease outbreaks are often associated with rainy seasons in the tropics [8].

Although *P. vivax* was more prevalent than *P. falciparum* in the studied area, CoRE with at least one pathogen was more frequent in patients with *P. falciparum*, which could be due to several factors including the origin of the patients, with *P. falciparum* predominating in the Sifontes municipality where most of the mineworkers come from, the occupation with the highest prevalence of CoRE. Unfortunately, there is no information available on the epidemiological behaviour of the studied pathogens in the study region, which does not allow conclusions to be drawn. On the other hand, a high frequency (42.9%) of complicated malaria was found and complications were more likely in CoRE patients compared to no CoRE ones, suggesting that CoRE with another pathogen could exacerbate the malaria clinical course. Nevertheless, as expected, there was a higher proportion of complicated cases in patients with *P. falciparum* than with *P. vivax* due to its higher pathogenicity, so this could be a confounding factor for the interpretation of these results. Further investigations are needed to confirm this observation due to the small sample size. Similar results have been found in patients with *P. vivax*/DENV co-infection, who were more likely to have severe disease than those mono-infected with DENV [32]. In contrast, Andrade et al. [10] found that HBV infection was associated with a lower intensity of malaria infection. To determine authentic association between co-infections, prospective studies should include a larger number of patients, although control of real-life variables remains a challenge in Venezuela.

The limitations of this study include the lack of double testing for viral infection due to economic reasons. Although we evaluated specific IgM antibodies, cross-reactivity cannot be ruled out due to polyclonal activation induced by *Plasmodium* infection [57, 58], as occurs with other highly prevalent infectious diseases, including that caused by Epstein Barr virus [59]. Another limitation was the absence of comparison between acute and convalescent sera from the same patient and the inability of molecular testing to confirm co-infection, since in some cases specific IgM remains positive for weeks after the acute phase [60–62]. Nonetheless, this is a frequent real-life situation regarding resources and poor settings where testing for follow-up of recovered patients is often not performed and where molecular diagnostic studies are restricted. Enrolment of only febrile individuals also constitutes a limitation; then future studies should include asymptomatic individuals or those with other viral/bacterial infections, but without malaria, to assess the real burden of co-infections in malaria and better predict their clinical outcome. Finally, the small number of CoRE patients, along with even smaller frequencies of some co-infections and several highly prevalent diseases that were not explored (e.g., Chagas disease, tuberculosis, leishmaniasis, human immunodeficiency virus, or syphilis), also represented a limitation.

## Conclusions

CoRE was confirmed in one third of the malaria patients and were more frequent in *P. falciparum*. The most frequent CoRE was DENV, followed by HAV, HBV, CHIKV, and LEP; HCV CoRE was absent. Complicated malaria was significantly more frequent in patients with CoRE than those without CoRE. The high prevalence of CoRE found in the main Venezuelan endemic state should contribute to the understanding of clinical and paraclinical behaviour to develop guidelines and protocols to optimise early diagnosis and guide treatment in patients with the acute febrile disease. Delay in the diagnosis or initiation of treatment of any of these infections could have fatal outcomes. Prospective studies with samples at different points of infection and the use of molecular tools are needed to clarify these findings.

## Supplementary Information

**Additional file 1: Figure S1. **Distribution of malaria cases according to the parasite species. (**A**) Map of Venezuela. (**B**) Origin of the malaria cases according to *Plasmodium* species (pie charts). Only main municipalities in Bolivar state are shown. Map also shows other relevant landscape features, including the localisation of the capital (asterisk), Ciudad Bolivar, in Heres municipality.

## Data Availability

All data generated or analysed during this study are included in the article.

## Abbreviations

DENV: Dengue virus
CHIKV: Chikungunya virus
LEP: Leptospirosis
HAV: Hepatitis A virus
HBV: Hepatitis B virus
HCV: Hepatitis C virus
IgM: Immunoglobulin M
IgG: Immunoglobulin G
ELISA: Enzyme-linked immunosorbent assay
CoRE: Co-infection or recent exposure
*OR*: Odds ratio
*CI*: Confidence interval
AST: Aspartate aminotransferase
ALT: Alanine aminotransferase
