# Supplementary material for: Seroprevalence of viral and bacterial pathogens among malaria patients in an endemic area of southern Venezuela
Source: Infect Dis Poverty. 2023 Apr 10;12:33. doi: 10.1186/s40249-023-01089-w (PMC10084699; doi:10.1186/s40249-023-01089-w)
Supplement: Supplementary file 1 — Additional file 1: Figure S1. Distribution of malaria cases according to the parasite species. (A) Map of Venezuela. (B) Origin of the malaria cases according to Plasmodium species (pie charts). Only main municipalities in Bolivar state are shown. Map also shows other relevant landscape features, including the localisation of the capital (asterisk), Ciudad Bolivar, in Heres municipality. [file 40249_2023_1089_MOESM1_ESM.docx]

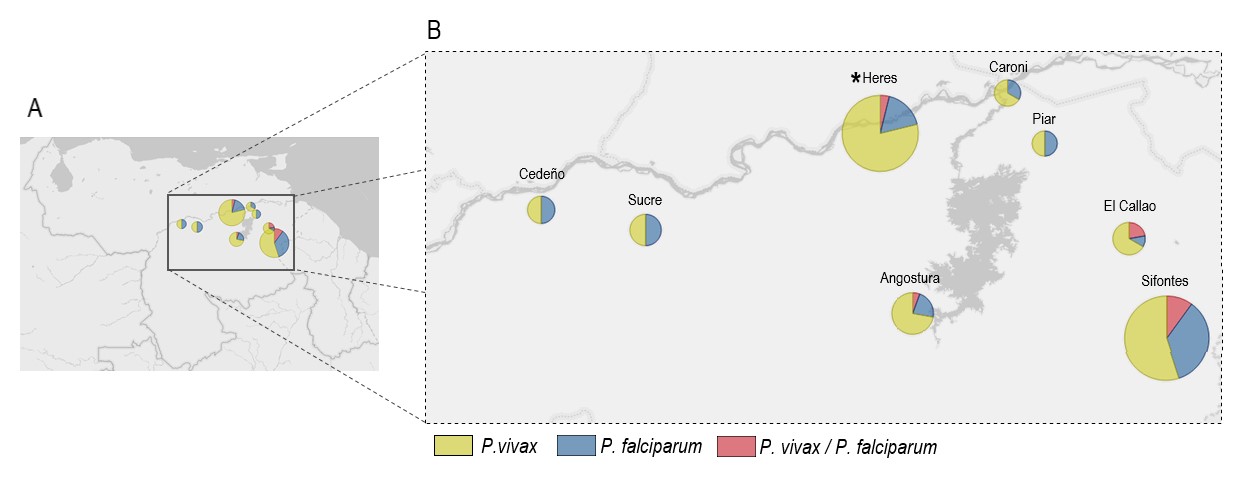


**Figure S1. Distribution of malaria cases according to the parasite species.** (**A**) Map of Venezuela. (**B**) Origin of the malaria cases according to *Plasmodium* species (pie charts). Only main municipalities in Bolivar state are shown. Map also shows other relevant landscape features, including the localisation of the capital (asterisk), Ciudad Bolivar, in Heres municipality.
